# Perception of University Nursing Students and Faculty Members Regarding Simulated Practices: A Mixed Methods Study

**DOI:** 10.3390/nursrep14040217

**Published:** 2024-10-14

**Authors:** Rosalbina Castillo Núñez, Rosa Nury Zambrano Bermeo, Nancy Francisca Bonilla Casierra, Massimo Tusconi, Felice Curcio, Cesar Ivan Aviles Gonzalez

**Affiliations:** 1 School of Health, Universidad Santiago de Cali, Cali 760001, Colombia; 2 University Hospital of Cagliari, 09042 Cagliari, Italy; 3 Faculty of Medicine and Surgery, University of Sassari (UNISS), Viale San Pietro 43/B, 07100 Sassari, Italy; 4 Faculty of Health Sciences Nursing Program, Universidad Popular del Cesar, Sede Sabanas, Valledupar 200002, Colombia; 5 Department of Medicine and Surgery, Nursing Faculty, University of Cagliari, 09124 Cagliari, Italy

**Keywords:** learning, perception, simulation training, students, nursing

## Abstract

Introduction: Clinical simulation has been used as a teaching strategy for students in health programmes, fostering greater preparedness and confidence in performing procedures. Objective: This study aimed to analyse the perception of fourth-semester nursing students and teachers regarding the simulated practice methodology implemented in a private university in Cali, Colombia. Method: A robust mixed-methods approach was used, incorporating quantitative surveys and qualitative interviews with 41 students and 5 teaching nursing faculty members. Data triangulation was applied to ensure the robustness of the results. Results: Both students and teachers reported a positive perception of simulated practice, which contributes to knowledge acquisition and contextual learning. Students emphasised that simulation improved their prior knowledge and motivated them to explore new topics. Lecturers emphasised the importance of well-trained instructors in simulation environments. However, participants identified challenges affecting performance, including simulation duration, group size, realism, and resource constraints. Conclusions: Students and teachers recommend strategic changes to the curriculum to optimise simulation practices.

## 1. Introduction

The academic performance of the student population serves as a crucial indicator of quality in higher education. We can observe this through the grades students obtain in their courses each semester. Academic performance represents the relationship between what students learn and what they achieve from a learning perspective [1]. Personal, social, and institutional factors influence this performance, determining students’ success or failure [1,2,3,4]. Thus, the teaching process must achieve positive academic performance, as evidenced by grades, and ensure significant learning. This means that students should grasp and contextualise concepts in real-world situations relevant to their professional roles [5,6]. Therefore, educators must also focus on developing students’ skills, competencies, and attitudes to meet the needs of their discipline [7,8].

To influence academic performance and meaningful learning, students in health programmes, as part of the theoretical–practical training process, begin clinical practice in simulated and natural environments. Simulation is a method that combines actual clinical activities with participatory guided interactive experiences [9,10]. Clinical simulation in nursing training offers numerous benefits, such as acquiring knowledge, building self-confidence, promoting teamwork, developing critical thinking skills, and creating a controlled and safe environment [11,12,13]. Therefore, educators must create clinical scenarios that closely resemble reality, facilitate active student participation, and encourage reflection so students can acquire the knowledge, skills, and competencies necessary to perform optimally in actual practice—providing high-quality and safe patient care [14,15].

Analysing the literature in contexts other than the Hispanic world, we can see that simulation-based education has proven to be a valuable tool for healthcare training, improving the acquisition of clinical knowledge and skills as well as ensuring patient safety. Görücü et al. (2024) [16] have demonstrated its effectiveness in improving the clinical decision-making skills of nursing students. Cho and Kim (2024) [17] suggest that simulation-based training can significantly improve nursing students’ overall empathy skills. Saragih et al. (2024) reported that scenario-based simulation courses have proven useful in increasing nursing students’ professional knowledge, clinical practice skills, and self-esteem in learning. Finally, Ismail et al. (2024) [18] emphasised that simulation-based education provides a risk-free environment for learning clinical practice while increasing patient safety.

In light of the above, the aim of this research is to explore the perceptions of teachers and students in the fourth semester of nursing regarding the quality of learning in simulated environments at a private university in Cali, Colombia.

## 2. Methods

### 2.1. Study Design

Between August 2021 and June 2022, a cross-sectional descriptive study with a mixed approach was conducted; a sequential quantitative–qualitative design was chosen as appropriate to explore the topic. A quantitative survey was followed by participant interviews (Table 1).

### 2.2. Participants Selection

Initially, 93 students enrolled in the fourth semester of the nursing programme of a private university in Cali, Colombia, were contacted. Participants were selected by convenience sampling. Of the 93 participants contacted, 41 students agreed to participate and fulfilled the inclusion criteria. Five teachers participated on the basis of their direct participation in simulation practices and teaching in the corresponding semester.

The sample included nursing students who were: (a) enrolled in the fourth semester of the nursing programme; (b) had participated in simulations and clinical practices; and (c) had provided consent for participation. For teachers, the inclusion criteria were (a) teaching in the fourth semester and (b) having conducted simulation activities during the study period. Table 1 describes the survey and interview samples and the variables addressed.

### 2.3. Simulation Sessions

The simulated practices developed at the university mainly used high-fidelity scenarios with manikins and standardised patients. In addition, on-site simulation sessions were conducted, in which students recreated clinical scenarios within the simulated hospital environment. These simulations were designed to replicate real-life situations that students might encounter during their professional practice, increasing realism and immersion. A total of six simulation sessions were conducted, each lasting approximately two hours. Each session had a maximum of ten participants working on solving specific clinical situations. The simulations covered topics such as surgical wound care, vital sign detection, aseptic technique, and patient communication. Each session ended with a structured debriefing, during which lecturers and students reflected on the procedures performed, evaluated errors, and discussed best practices and areas for improvement. This debriefing was conducted using the three-step feedback model: description of the procedure, analysis of actions, and conclusions on improvement.

### 2.4. Tools

To collect the study variables, two surveys were created, one for students and one for nursing teachers (Supplementary File S1). The questionnaires used were validated by a panel of four health science experts with experience in clinical simulation and teaching and with experience in qualitative and quantitative research. Pilot tests were conducted to ensure the content validity and internal consistency of the questions.

The construction of the instrument used was based on an exhaustive literature review, considering several studies on clinical simulation. Juguera Rodríguez et al. [19] analysed students’ perceptions of simulation as an effective pedagogical tool in nursing skills training. Amador and Bernal [20] highlighted the fundamental role of simulation in nursing education. Other studies, such as that of Angulo Mendoza et al. [21], evaluated the impact of virtual laboratories on learning technical skills. Barrios Araya et al. [22] showed how simulation improves students’ self-efficacy and locus of control. Caballero et al. [23] and De la Horra Gutiérrez [24] highlighted the usefulness of software and simulation for skills assessment. Finally, Valencia et al. [25,26] examined its effect on the development of critical thinking.

#### 2.4.1. Perception of Simulated Practices

The questionnaire used to assess perceptions of simulated practices consisted of 16 items for teachers and 14 items for students.

A 4-point Likert scale assessed teachers’ and nursing students’ perceptions of the factors and elements influencing their performance and the learning process in simulated practice. The response options of the Likert scale differed according to the item assessed (Supplementary File S1). For example, in the case of the questions assessing aspects of simulated practice, 1 = Insufficient; 2 = Insufficient; 3 = Good; 4 = Very Good.

The questionnaire assessed various aspects of the simulated practices, including their contribution to learning, the clarity of the procedures, the teaching support, the duration of the sessions, the realism of the scenarios, and the adequacy of the resources used. The items also assessed whether the teacher allowed the students to repeat the procedures until they learnt them and whether the students felt that the simulated practices adequately prepared them for real clinical practices. In addition, the questionnaire collected several socio-demographic variables, such as age, gender, marital status, socio-economic stratum (from 1 to 5, according to the National Administrative Department of Statistics (DANE) classification used in Colombia) [27], whether they had children and the number of children, and previous work experience and sector.

#### 2.4.2. Semi-Structured Interviews

This type of interview was chosen because it is particularly informative, allowing the researcher to create a framework for the topics discussed. Furthermore, a semi-structured interview guide (Supplementary File S2) was developed as it provides a clear set of instructions for interviewers and, at the same time, can provide reliable and comparable qualitative data [28]. Finally, enquiries and clarifications were requested during the interviews to ensure complete data information was obtained [29].

After reading the relevant literature, the researchers developed 2 semi-structured questionnaires, one for students and the other for teachers. The students’ semi-structured interview consisted of 5 questions, which focused on exploring their experience and perceptions regarding simulated and actual practices in the academic environment. They were also asked about aspects such as the realism of simulation, the effectiveness of learning, and their preparedness for clinical practice.

The interview with the lecturers consisted of 5 questions that collected information on their experience in teaching through simulation, perceived effectiveness of simulated practices, and challenges faced during teaching.

### 2.5. Data Collection Procedures

The participants were contacted directly by the university, and prior to the start of the study, there was no direct relationship between the researchers and the participants.

To ensure the accuracy and reliability of the data collection, training sessions lasting approximately 20 min were conducted. These were designed to guide the students in the correct completion of the questionnaire and to emphasise the importance of honest and accurate answers and the importance of their participation in the improvement of simulation-based education. The participating students were informed about the objectives, methods, and procedures of the study, with a strong emphasis on voluntary participation and anonymity. Furthermore, it was emphasised that refusal to participate would have no negative consequences on their academic status or any other aspect.

The surveys were provided during live meetings at the end of the simulated practices by 2 researchers (RCN and NFBC) and took approximately 10-15 min to complete. During the data collection period, the researchers were available to answer any requests for clarification. Both researchers (RCN and NFBC) had extensive experience and training in qualitative research, particularly in conducting semi-structured interviews and thematic analysis. This background ensured that the interviews were conducted in a rigorous manner and that the data collected was reliable and accurate.

Semi-structured interviews were conducted in designated rooms within the university, providing a comfortable and confidential environment to encourage participants to express themselves freely. Each interview was conducted face-to-face and on an individual basis. The average duration of each interview was 40 min. The interviews were audio-recorded and transcribed in full to ensure the accuracy of the information and the confidentiality of the participants was always maintained.

### 2.6. Statistical Analysis

#### 2.6.1. Quantitative Analysis

A descriptive analysis of the variables was performed, expressing the qualitative variables in frequencies and percentages and the quantitative variables in means and/or standard deviations. In addition, bivariate analyses were conducted to identify significant associations between socio-demographic variables and students’ perceptions of simulated practices. The distribution of responses was interpreted according to crucial aspects of learning and the perceived effectiveness of simulations. All analyses were carried out using the IBM© SPSS Statistics v.22.0 statistical programme.

#### 2.6.2. Qualitative Analysis

The semi-structured interviews were transcribed in full, assigning an identification code to each one, and a thematic analysis of the qualitative data was conducted. The analysis was conducted inductively, allowing the identification of patterns and emerging themes relating to students’ and teachers’ perceptions of simulation practices. Responses were coded and organised into categories such as “simulation realism”, “pedagogical effectiveness”, and “implementation challenges”. These categories were compared between student and teacher responses, looking for similarities and differences.

#### 2.6.3. Integration of Quantitative and Qualitative Analysis

A triangulation approach was used to integrate the results of the quantitative and qualitative analysis. The quantitative survey results provided an overview of the perception of simulated practices, while the qualitative data offered a deeper understanding of the experiences of individual students and teachers. Triangulation allowed the results to be corroborated and enriched the final interpretation, highlighting areas of convergence, such as the perceived importance of simulations for professional development, and areas of divergence, such as the limits of realism and time allocated to the practices.

## 3. Results

### 3.1. Sociodemographic Characterisation of Students

The minimum age of the participants was 18 years and maximum 41 years, with a mean of 22.6 (SD 4.6) years; 56.1% of the students were between 18 and 22 years old, followed by the 23–27 age group with 32.2%. Regarding gender distribution, 85.4% were women. In total, 80.5% are single, and 12.2% said they were married. About distribution by socioeconomic stratum, 48.8% are in stratum 3 and 14.6% in stratum 1. Regarding the number of children, 85.4% said they did not have them (Table 2).

### 3.2. Student Assessment of Aspects of Simulated Practices

Table 3 summarises the students’ responses to various aspects of simulated practices, such as duration, clarity, realism of procedures, teaching support, and clarity and complexity of clinical cases. A Likert-type scale was used, from 1 to 4, where 1 is insufficient and 4 is very suitable. Overall, 73.1% of students stated that they had a good theoretical foundation, and 19.5% stated that it was sufficient. Likewise, the students were asked about the aspects of the simulated practices, such as the duration of the practice, the clarity and realism of the procedures carried out, the teaching support, and the clarity and complexity of the clinical cases, which affect the perception that the student has about this kind of teaching strategy.

Regarding the elements that influence students’ performance and learning in simulated practice, most students rated the different aspects positively as ‘good’. The clarity of clinical procedures was highly rated as ‘good/very suitable’ by 93.1% of students. Likewise, high ratings were achieved for teaching support (80.2% good/very sufficient), complexity of clinical cases (87.8% good/very sufficient), and realism (85.3% good/very sufficient).

Furthermore, the methodology, duration, and complexity were rated. The aspects that showed the most student dissatisfaction were the time allocated and the number of practices scheduled per semester. One-fifth of students (19.5%) stated that simulated practice almost never prepares them for real-world practice.

Students were asked to evaluate various aspects of simulated practices based on their experience and perception. While 7.3% felt that simulated practice rarely contributed to academic performance and 12.2% indicated that the simulated hospital was rarely well equipped to meet course objectives, most students had more positive perceptions. For example, 7.3% reported that the time taken by the lecturer to explain the procedures was sufficient. A total of 88% of the students stated that the length of the simulation was sufficient and/or always sufficient. Likewise, 80.5% felt that the lecturer repeated and/or always repeated the procedures sufficiently. Finally, 19.5% of students also felt that simulation rarely prepared them for real-world practice.

On the other hand, considering the level of demand, most students perceived the methodology, clinical cases, evaluation methods, and environment as demanding or very demanding (65.9%). However, 2.4% indicated there was little demand on them regarding the evaluation, theoretical foundation, and setting, and another 2.4% stated that the evaluation methods are reasonable.

### 3.3. Sociodemographic Characterisation of Teachers

Likewise, a survey was conducted with teachers to find out their perceptions of simulated practices. Five teachers in charge of teaching this class were consulted for this purpose.

The sociodemographic characterisation of the distribution by age found a minimum age of 27 and a maximum age of 67, with a mean of 48.4 (SD 18.8) years. Regarding the distribution by gender, 100% corresponded to women; 40% are married, and the same percentage are single. A total of 40% reside in stratum 4, and the same percentage reside in stratum 5. Work experience can influence teaching performance. They stated that they had a minimum of one year and a maximum of thirty years of teaching experience. Regarding experience teaching simulated practice, 20% stated they had one year of experience, and 80% had four years.

Relevant aspects of the simulated practices were consulted, particularly regarding previous experience and training; 100% of the teachers report having no previous experience before beginning to teach this type of lecture at the university. A total of 40% reported that the university had yet to train them to teach in simulated environments; however, 80% were interested in accessing training soon.

### 3.4. Teacher Assessment of Aspects of Simulated Practices

Considering that the simulated practice comprises different elements that students and teachers perceive, the teachers were asked whether they considered these aspects appropriate. A Likert-type scale was used, from 1 to 4, where 1 is insufficient and 4 is very suitable. As seen in Table 4, 100% considered the time or length of the practice, clarity of the procedures carried out, and teacher support as very suitable, and 80% considered the complexity and realism of the procedures in the same way. Regarding these last two aspects, there is evidence of a possibility for improvement.

Further elements evaluated were associated with themes, methodology, duration, and complexity of procedures. The responses revealed opportunities for improvement: 20% of teachers considered that the topics were rarely discussed in depth, another 20% considered that the simulated environment does not facilitate understanding of the procedures, and 40% considered that the student is rarely able to achieve mastery of the topic. Regarding the duration of simulation practice, 40% indicated that there is rarely enough time, 20% stated that there is never enough time to explain the use of simulation equipment, and 20% considered that the simulated environment is never realistic. In addition, 60% considered that the distribution of students per teacher rarely allows the teacher to repeat the procedures to improve learning.

One of the aspects that teachers were asked to evaluate was the demand for simulated practices; emphasis was placed on the methodology, the clinical cases addressed, the evaluation method, the theoretical foundation, and the setting of each procedure. Regarding the methodology used, 60% consider it demanding and 40% very demanding. Regarding the cases presented, 20% consider them undemanding, 40% consider the evaluation methods in the same way, 20% the theoretical foundation, and 40% the setting of each activity; each aspect has a potential for improvement, as indicated by the teachers.

### 3.5. Analysis of Simulated Practice Interviews

An interview was conducted with the students and teachers participating in this research, in which the importance of this type of practice, the difficulties and challenges of the learning strategies, the difficulty of evaluation, teaching qualifications, methodology, inputs, instruments, and level of difficulty were addressed. Table 5 summarises the main findings for each category evaluated for students and teachers. Q1, Q2, Q3, Q4, and Q5 correspond to the questions of the semi-structured interview for teachers and students. All the questions in this paper are provided in Supplementary File S2.

Despite the negative aspects, students and teachers emphasised the need to continue and strengthen simulated practices because they allow students to minimise fear before contact with real practices. They also allow them to perform different procedures with the option of making mistakes without putting the patient at risk. The latter is an issue that students value, given that they learn from mistakes, so they also insisted on allocating more time to the simulation to repeat the procedures.

### 3.6. Data Triangulation

From the surveys and interviews, similarities in the perception of various aspects were evident, such as the importance of the simulated practice, demand, and evaluation methods. In the union of these aspects, similarity was found in some factors, as presented in Table 6, which allows us to visualise more clearly the positive aspects and challenges faced by simulated practices.

### 3.7. Integration Analysis: Commonalities and Disagreements between Teachers and Students

In the qualitative analysis, several commonalities and disagreements were identified between teachers and students on perceptions of simulated practices.

Commonalities:

1. Importance of simulated practices: Both teachers and students emphasised the value of simulated practices in preparing students for real-world clinical environments. Both groups agreed that these practices help reduce anxiety before interacting with real patients and reinforce theoretical knowledge.
  ○ ‘*Simulated practices help us feel prepared for real-world situations*’ (Student 5)
  ○ ‘*These practices introduce students to the hospital environment and strengthen their clinical reasoning*’ (Teacher 2)
  ○ ‘*Simulated practices help us feel better prepared to face real situations in the hospital. I feel safer practising without putting a patient at risk*’ (Student 13)
  ○ ‘*These practices allow students to understand the hospital environment before having direct contact with patients, which reduces their anxiety*’ (Teacher 1)
2. Simulation length: Both students and teachers expressed concern about the insufficient time allowed for each simulated practice. They agree that the limited time limits students’ ability to repeat procedures and receive personalised feedback.
  ○ ‘*We don’t have enough time to repeat procedures and improve*’ (Student 7)
  ○ ‘*The time is too short for each student to fully develop the necessary skills*’ (Teacher 3)
  ○ ‘*Time is very limited. Sometimes, we don’t have enough time to repeat the procedures several times so that we can become more confident in practice*’ (Student 27)
  ○ ‘*The number of students per teacher makes it difficult to provide individual feedback and ensure that everyone has correctly understood the procedures*’ (Teacher 2)
3. Availability of equipment: Both groups identified limitations in the availability and realism of simulation equipment. Students and teachers recognised that the lack of adequate equipment affects the quality of the simulated experience.
  ○ ‘*The equipment is rarely similar to what we would use in a real hospital*’ (Student 19)
  ○ ‘*Simulated equipment needs to be more realistic for effective learning*’ (Teacher 4)
4. Evaluation methods: Both students and teachers recognised difficulties in the evaluation methods used during the simulated exercises.
  ○ ‘*In the assessments, I sometimes had the feeling that there is too much focus on theoretical knowledge and not enough on how to apply that knowledge in practice*’ (Student 40)
  ○ ‘*Assessing each student’s practical performance in a simulated context can be difficult, especially when there is little time to conduct in-depth evaluations*’ (Teacher 5)

Disagreements:

1. Realism of simulated practices: While teachers believe that the simulation process is sufficiently realistic to support the learning objectives, students are more critical of the lack of realism, particularly with regard to emotional interaction with patients.
  ○ ‘*Simulations do not fully prepare us for the emotional aspects of patient care*’ (Student 10)
  ○ ‘*The simulation is realistic enough to introduce students to real clinical scenarios*’ (Teacher 1)
2. Focus of assessments: Teachers tended to emphasise the importance of theoretical knowledge in their assessments, while students felt that there was too much emphasis on theory at the expense of practical application.
  ○ ‘*Assessments focus too much on theory and not enough on practice*’ (Student 36)
  ○ ‘*A strong theoretical basis is essential to apply practical skills correctly*’ (Teacher 5)

The integration of these results highlights key areas where teachers and students share similar concerns, such as time constraints and equipment availability. However, important differences were found in their views on the realism of simulations and the purpose of assessments.

The length of simulation practices is a variable with different implications. Not all students can perform the different procedures, which compromises the perception of the experience. Likewise, this limits the teacher’s evaluation, covering dimensions such as being, doing, and knowing. Furthermore, time limits a student’s ability to repeat a procedure to correct possible errors or shortcomings.

Although teachers and students believe that simulated practice allows them to strengthen theoretical knowledge, criticism was evident from teachers regarding the theoretical bases and the attitudes of students who need to be prepared and informed to address the clinical cases presented. For their part, the students expressed the importance of preparing through the guides provided to them before each practice; however, they mentioned that the semester’s academic overload means they have little time to develop them.

Regarding the difficulties around simulated practice when using it as a pedagogical strategy in the training of fourth-semester nursing students, a critical issue is the possible realism of the setting of each clinical case. On the one hand, the equipment does not always recreate the same conditions of a hospital, and on the other, there is no culture of simulation, which prevents the student from being immersed in an identical recreation and leads to them laughing at certain behaviours or downplaying their importance. Despite these difficulties, teachers and students highlight the benefits of simulated practice. During the interviews, possible recommendations were formulated, such as: better equipping the simulated hospital with equipment that has better features and inputs; starting with the simulation from the first semester; increasing the duration of each practice; and reducing the groups so that the teacher has greater availability for each student. Even a proposal from two students, which caught our attention, was to ensure that the simulated hospital was ready for voluntary consultation so that it could be accessed with the accompaniment and guidance of the higher semester monitors.

## 4. Discussion

The results suggest that simulated practice may improve students’ academic performance by enabling them to internalise concepts, contextualise them, and reflect on their experiences, which may contribute to the development of the skills and competencies required to carry out procedures and activities related to the care of persons with altered health status. In the present investigation, it emerged that the simulation was articulated in such a way as to introduce students to clinical practice, which becomes the setting in which the knowledge learnt in the previous semesters converges.

According to Fernandez-Quiroga et al. (2017) [30], the use of simulation follows a structured approach in which resources are aligned with the educational process and are supported by clearly defined learning objectives and a predetermined methodology. This is consistent with the findings of the present research, in which simulated practice for nursing students is integrated as part of an institutional strategy. With this strategy, the ‘simulated hospital’ is incorporated into the curriculum, and teachers are specifically trained to teach in this environment. However, the need for continuous institutional updating of pedagogical practices has also been identified.

When reviewing positive aspects of the simulated practice, teachers and students agreed that the simulated environment strengthens the theoretical foundation because it allows for verification and deeper understanding [22,31,32]. Similarly, simulation encourages reflection on the topics addressed, such as the hospital environment; in the latter, the teacher plays a crucial role because, through their experience, they enable each student to broaden their perspective on the planning of comprehensive, knowledge-based, and critical thinking nursing care.

The students acknowledged that the simulated practice reinforced previously learnt knowledge but also motivated them to investigate new topics. This is an obvious benefit that is maximised with the support of teachers as they contribute their experience to broaden topics that should be considered in the development of healthcare institutions, particularly in approaching the patient through assertive communication [33]. These benefits agree with other research that mentions the importance of simulation as a pedagogical strategy in the health sciences educational process [34] and that simulated practice should be articulated within a pedagogical strategy, where benefits and challenges are considered to continually strengthen it [35].

The inclusion of simulated practice positively modifies the vision of the teacher–student relationship, given that traditionally, the teacher is the one who guides the process and imparts knowledge. Simulation allows the student to learn from the process, from their own experience, including their errors [19]. In this research, it was found that there is an orientation towards the self-structuring of knowledge, with the teacher being a facilitator who corrects, explains in more detail, contextualises each concept, and contributes from their experience.

Simulated practice also leads to generating meaningful student experiences [25]. Furthermore, the dynamics of interaction during each clinical case, as well as the teachers’ guidance, lead to generating motivation and learning in each student, generating confidence that is reflected in their attitude and ability to make decisions. In line with our results, Perdomo-Martínez et al. (2022) [36] state that simulation is essential to increase quality, ensure safety, and increase confidence in performance, as well as in the acquisition of skills and knowledge, provided it is applied in realistic environments that promote critical reasoning and decision-making.

The success of simulation depends on the existence of high physical fidelity, in which manual skills, clinical reasoning, and problem-solving skills are developed, and, finally, high emotional or experiential fidelity, in which the retention of information through the management of complex processes involving knowledge or emotions is fostered [37]. However, although realism in this type of simulation brings theory closer to practice, it does not replace the clinical hospital field.

The above led us to reflect that simulated practice must be articulated from the first semester so that the student becomes accustomed to using information and communication technology (ICT) tools as part of the pedagogical process and teaching strategy. According to Angulo Mendoza et al. (2012) [21], using simulated environments favours reflection and allows students to advance according to their learning pace. In this same sense, Urra Medina et al. (2017) [38] consider that the use of simulation must obey an educational model that integrates different teaching strategies, and these should not be limited to one semester; the dynamics of professional training must be valued so that from the first semester there is an approach to the student using virtual or simulated environments [38]. The students consulted suggested that this type of tool be used from the first semester so that there is familiarisation and culture about the use of simulation and the role it must fulfil.

It is important that the interaction with the patient is simulated during practice so that the student’s experience is made as realistic as possible.

According to the teachers, the patient’s communication and emotional expression are topics that the students don’t cover in the simulated practice because they are unable to project or imagine the patient in each exercise performed.

### Study’s Limitations

The study has some limitations. First, although the sample size may be perceived as small, the sequential quantitative–qualitative method is the best option to explore the perceptions of different target groups, and qualitative samples are not required to be large. The results should be generalised to other educational contexts with some caution. Second, the study was conducted in a single private university, while conditions may vary in other universities, leading to different responses from teachers and students. Third, we noted the presence of limited simulation resources, hindering the experience of both groups. Furthermore, this study does not allow for a longitudinal assessment of the impact of simulations on clinical performance. This may have provided a more comprehensive view of the effectiveness of the simulation pedagogical methodology.

## 5. Conclusions

Both students and teachers have a positive perception of simulated practices in the nursing training process, and it is evident that this contributes to strengthening the acquisition of knowledge through contextualisation. Furthermore, it allows the student to overcome fears or apprehensions towards practical procedures; simultaneously, it becomes a favourable scenario for introduction to the clinical environment. Likewise, other benefits were highlighted, such as the strengthening of prior knowledge and the stimulation towards research into new topics associated with the nurse’s practice in dealing with patients’ care.

Students and teachers agree on the challenges and limitations of current simulated nursing practices. Issues such as duration, the number of students assigned per group, and the lack of supplies affect the performance of the different procedures. Despite the above, these shortcomings can be overcome in the short term by restructuring the planning of this type of practice in accordance with the curriculum and institutional projects.

The findings showed that the teaching strategy based on simulated practices that are applied to fourth-semester nursing students seeks to strengthen the theoretical knowledge that they learnt in previous semesters, which is developed through clinical cases. This invites students to apply their acquired knowledge while promoting the investigation of new topics. The strategy of simulated practices allows the theoretical and practical to converge while introducing the student to the hospital environment, thus promoting research on patient diagnoses (underlying disease), care protocols, and medication administration, among other procedures, which must be conducted with theoretical argumentation and reflective analysis. As reported by the students, this inquiry competence is promoted by proactive teachers who, with their expertise, motivate the development of professional knowledge and skills.

## Figures and Tables

**Table 1:** List of techniques, samples, and instruments.

Technique	Sample	Instrument	Variables/Categories You Address
Survey	41 students 5 teachers	A survey questionnaire with a Likert scale was used to evaluate implicit factors in practice.	Scheduled activities. Theoretical foundations. Several activities developed. Practice or exercise environment. Teacher support. Achievements to evaluate. Evaluation methodology. Time per activity.
Interview	41 students 5 teachers	Format	Simulated practical importance. Difficulties with learning strategies. Pedagogical strategy difficulties. Pedagogical strategy challenges. Evaluation difficulties. Teaching qualification. Teaching methodology. Supplies and instruments. Difficulty level.

Source: own elaboration.

**Table 2:** Sociodemographic classification of students.

	Mean (SD)	n.	Percentage
Age	22.6 (4.6)		
Between 18 and 22 years		24	56.1%
Between 23 and 27 years		13	32.2%
More than 28 years		4	9.8%
Gender			
Female		36	85.4%
Male		6	14.6%
Civil status			
Does not inform		1	2.4%
Married		5	12.2%
Single		33	80.5%
Free union		2	4.9%
Socioeconomic			
Stratum 1		6	14.6%
Stratum 2		10	24.4%
Stratum 3		20	48.8%
Stratum 4		4	9.8%
Stratum 5		1	2.4%
Number of children			
Does not have		35	85.4%
One		5	12.2%
Two		1	2.4%

Source: own elaboration.

**Table 3:** Students’ assessment of simulated practice aspects.

	Insufficient	Sufficient	Good	Very Suitable
	n (%)	n (%)	n (%)	n (%)
Theoretical foundation	2 (4.9%)	8 (19.5%)	30 (73.1%)	1 (2.4%)
Length of practice	1 (2.4%)	2 (4.9%)	29 (70.7%)	9 (21.9%)
Clarity of the clinical cases	1 (2.4%)	3 (7.3%)	29 (70.7%)	8 (19.5%)
Clarity of procedures	(0%)	2 (4.9%)	31 (75.6%)	8 (19.5%)
Teaching support	1 (2.4%)	3 (7.3%)	30 (73.1%)	7 (17.1%)
Complexity of clinical cases	2 (4.9%)	3 (7.3%)	26 (63.4%)	10 (24.4%)
Realism of procedures	2 (4.9%)	4 (9.8%)	27 (65.9%)	8 (19.5%)

Source: own elaboration.

**Table 4:** Teachers’ assessment of aspects of simulated practice.

Aspects	Insufficient	Sufficient	Good	Very Suitable
	n (%)	n (%)	n (%)	n (%)
Time (length) of practice	0 (0%)	0 (0%)	0 (0%)	5 (100%)
Clarity of the procedures	0 (0%)	0 (0%)	0 (0%)	5 (100%)
Teacher support	0 (0%)	0 (0%)	0 (0%)	5 (100%)
Complexity of procedures	0 (0%)	0 (0%)	1 (20%)	4 (80%)
Realism of the procedure	0 (0%)	0 (0%)	1 (20%)	4 (80%)

Source: own elaboration.

**Table 5:** Consolidated interview results.

Ask	Category	Students	Teachers
Q1.	Importance of simulated practice	Prepare for real practice. Reduce fear of practice. Introduces the student to the hospital environment. Reaffirms theoretical knowledge. Expands the theoretical bases and foundations. Promotes research on new topics addressed in clinical cases. Allows the student to learn from mistakes. Minimises the risk derived from errors.	Introduces the student to the hospital environment. Lets the student know the “step-by-step” approach to the patient. Reaffirms and deepens the theoretical bases. Expands knowledge by contextualising it with the patient’s diagnosis.
Q2.	Difficulties in the learning strategy	The time allocated to each practice. The number of students per practice. Low interaction with teams. Weak realism and setting of clinical cases.	Too many students are assigned per teacher. The time allocated for practice. Training in the use of the equipment.
Q3.	Pedagogical strategy difficulties	Lack of realism due to the emotional dimension of the patient. Practice time; not all students can perform the procedures. Equipment limits specific exercises. Lack of supplies that limit the execution of procedures.	There needs to be more realism throughout the simulated practice. The student does not commit to or assimilate the simulation. Time limits the strategy and teaching work.
	Pedagogical strategy challenges	Increase practice time for each student to perform the procedure. Start with simulated practices from the beginning of the semester and/or the beginning of the degree.	Reduce the number of students assigned per teacher. Increase the time of simulated practices.
Q4.	Evaluation difficulties	The teacher focuses on knowledge, not the teacher’s attitude or motivation. Not all students perform the procedures; therefore, practical performance is not graded.	The time and students assigned limit the teacher’s ability to focus on individual performance and assess different elements of the experience.
Q5.	Teaching qualification	The teachers are prepared. The teachers contribute from their experience. The teachers have experience and allow the student to expand their knowledge.	The qualification is adequate, but induction and training on the simulation equipment and instrument are required.
	Teaching methodology	It is adequate, excellent, very good. The teacher explains using their knowledge and experience. The teacher clarifies doubts as they arise. The teacher is creative in explaining doubts or new topics.	Each teacher uses their methodology. In teaching practice, theoretical aspects are deepened. The limited time of practice limits the methodology.
	Supplies and instruments	Inputs are missing. The student must purchase supplies. The lack of supplies compromises the execution of the procedures. Due to a lack of resources (money), the student cannot purchase supplies.	There is a lack of induction and training for the use of simulators. The allocation of supplies and time must be improved so that each student can perform the procedure.
	Difficulty level	The practice is demanding. There are many differences between actual practice and real practice due to patient interaction. The setting and realism of the simulated practice must be improved.	More setting and realism are required for the student to become involved. Improve simulators that allow greater mobility and realism.

Source: own elaboration.

**Table 6:** Data triangulation: students and teachers.

General Features	Factors	Students Survey	Students Interview	Teachers Interview
Simulated Practice Importance	Simulated practice importance	51% always contribute to professional training	Prepares the student for real practice in the hospital environment	Introduces students to the hospital environment Strengthens students’ clinical reasoning skills
Simulated Practice Attributes	Theoretical foundation	73.2% adequate	56% allow the application of theoretical foundations Strengthens the theoretical foundation	Theoretical foundation is essential for the practice Teachers believe that students need more theoretical reinforcement
Teaching Qualification	Teacher support	88% very adequate	Students positively perceive the preparation and experience of teachers	Teachers feel their preparation is adequate, but training in simulation is needed
Teaching Methodology	Teaching methodology	66% consider it demanding, 34% very demanding	Adequate; teachers make an effort to explain and clarify doubts	Teachers consider the methodology demanding but realistic
Time or Length of Practice	Length of simulated practice	88% very adequate	Students positively perceive the preparation and experience of teachers	Teachers feel their preparation is adequate, but training in simulation is needed
Supplies and Instruments	Equipment availability	12% consider that they are rarely adequately equipped	Students are critical of the lack of supplies and materials	Teachers express concerns about inadequate equipment for simulation
Simulated Practice Requirements	Learning strategy difficulties	56% consider that what has been learned is constantly evaluated	Focuses too much on theoretical learning, lacks practical application	Teachers agree that assessments focus on theory, neglecting practical performance
Pedagogical Strategy Difficulties	Realism of simulation equipment	80% very adequate realism, and 15% feel equipment is rarely realistic	Simulated environment lacks realism	Teachers agree the equipment does not always reflect real-life conditions
Pedagogical Strategy Challenges	Preparation for real practice	20% consider it rarely prepares them for real practice	Lack of realism affects student readiness for real clinical practice	Teachers believe that while simulation is useful, it does not fully prepare students
Evaluation Methods	Evaluation difficulties	59% consider it demanding	Evaluation focuses on theory, not practical skills	Teachers note that student motivation and practical performance are not sufficiently evaluated
Difficulty Level	Complexity of clinical cases	66% very appropriate	Students find the clinical cases challenging, but the lack of realism hinders learning	Teachers find the clinical cases demanding but acknowledge the limitations in realism

Source: own elaboration.

## Data Availability

All data generated or analysed during this study are included in this published article.

## Supplementary Materials

The following supporting information can be downloaded at: https://www.mdpi.com/article/10.3390/nursrep14040217/s1, Supplementary File S1: Survey; Supplementary File S2: Semi-structured interview guide.

## Public Involvement Statement

No public involvement in any aspect of this research.

## Guidelines and Standards Statement

This manuscript was drafted against the Mixed Methods Reporting in Rehabilitation & Health Sciences (MMR-RHS) for Mixed Methods research https://www.equator-network.org/wp-content/uploads/2023/09/MMR-RHS-checklist.pdf (accessed on 11 October 2024).

## References

[1] Vargas G.M.G. (2007). Factores asociados al rendimiento académico en estudiantes universitarios, una reflexión desde la calidad de la educación superior pública. Rev. Educ..

[2] Erazo-Santander O.A. (2012). El Rendimiento Académico, Un Fenómeno de Múltiples Relaciones y Complejidades. Rev. Vanguard. Psicológica Clínica Teórica Práctica.

[3] Alfonso J., Martínez J. (2015). Modelos de Simulación Clínica Para La Enseñanza de Habilidades Clínicas En Ciencias de La Salud. Rev. Mov. Cient..

[4] Cedeño M.S.A., Pérez M.T.G., Venegas M.d.L.C. (2015). Análisis de Los Factores Que Contribuyen al Éxito Académico En Estudiantes Universitarios: Estudio de Cuatro Casos de La Universidad de Colima. Rev. Int. Educ. Aprendiz..

[5] Ghezzi J.F.S.A., Higa E.d.F.R., Lemes M.A., Marin M.J.S. (2021). Strategies of Active Learning Methodologies in Nursing Education: An Integrative Literature Review. Rev. Bras. Enferm..

[6] HadaviBavili P., İlçioğlu K. (2024). Artwork in Anatomy Education: A Way to Improve Undergraduate Students’ Self-Efficacy and Attitude. Anat. Sci. Educ..

[7] Xia Y., Huang H., Halili X., Wang G., Chen Q. (2024). Development of an Evidence-Based Nursing Practice Course Framework for Undergraduate Nursing Students from a Perspective of Academic-Practice Partnerships: A Delphi Study. Nurse Educ. Pract..

[8] Contreras-Ramos L.M., Laguado Jaimes E., Jaimes Carvajal N.E., Pico Ferreira M., Villamizar-Osorio M.L. (2024). Socioemotional Skills in the Teaching-Learning Process Mediated by Medium- and High-Fidelity Clinical Simulation in Nursing Students: Protocol for a Scoping Review. JMIR Res. Protoc..

[9] Foronda C.L., Fernandez-Burgos M., Nadeau C., Kelley C.N., Henry M.N. (2020). Virtual Simulation in Nursing Education: A Systematic Review Spanning 1996 to 2018. Simul. Healthc. J. Soc. Simul. Healthc..

[10] Rim D., Shin H. (2021). Effective Instructional Design Template for Virtual Simulations in Nursing Education. Nurse Educ. Today.

[11] Dawood E., Alshutwi S.S., Alshareif S., Shereda H.A. (2024). Evaluation of the Effectiveness of Standardized Patient Simulation as a Teaching Method in Psychiatric and Mental Health Nursing. Nurs. Rep..

[12] Medel D., Reguant M., Cemeli T., Jiménez Herrera M., Campoy C., Bonet A., Sanromà-Ortíz M., Roca J. (2024). Analysis of Knowledge and Satisfaction in Virtual Clinical Simulation among Nursing Students: A Mixed Study. Nurs. Rep..

[13] Moreno-Cámara S., da-Silva-Domingues H., Parra-Anguita L., Gutiérrez-Sánchez B. (2024). Evaluating Satisfaction and Self-Confidence among Nursing Students in Clinical Simulation Learning. Nurs. Rep..

[14] Amaro-López L., Hernández-González P.L., Hernández-Blas A., Hernández-Arzola L.I. (2019). La simulación clínica en la adquisición de conocimientos en estudiantes de la Licenciatura de Enfermería. Enferm. Univ..

[15] Moreno G., Meneses-Monroy A., Mohamedi-Abdelkader S., Curcio F., Domínguez-Capilla R., Martínez-Rincón C., Pacheco Del Cerro E., Mayor-Silva L.I. (2024). Virtual Active Learning to Maximize Knowledge Acquisition in Nursing Students: A Comparative Study. Nurs. Rep..

[16] Görücü S., Türk G., Karaçam Z. (2024). The Effect of Simulation-Based Learning on Nursing Students’ Clinical Decision-Making Skills: Systematic Review and Meta-Analysis. Nurse Educ. Today.

[17] Cho M.-K., Kim M.Y. (2024). Effectiveness of Simulation-Based Interventions on Empathy Enhancement among Nursing Students: A Systematic Literature Review and Meta-Analysis. BMC Nurs..

[18] Ismail F.W., Ajani K., Baqir S.M., Nadeem A., Qureshi R., Petrucka P. (2024). Challenges and Opportunities in the Uptake of Simulation in Healthcare Education in the Developing World: A Scoping Review. MedEdPublish.

[19] Juguera Rodríguez L., Díaz Agea J.L., Pérez Lapuente M.L., Leal Costa C., Rojo Rojo A., Echevarría Pérez P. (2014). La Simulación Clínica Como Herramienta Pedagógica. Percepción de Los Alumnos de Grado En Enfermería En La UCAM. Enferm. Glob..

[20] Amador A.R., Bernal B.M.L. (2017). La Simulación En La Enseñanza de La Enfermería. Rev. Fac. Med. UNAM.

[21] Angulo Mendoza G.A., Vidal Espinosa L.O., García Ortiz G. (2012). Impacto del laboratorio virtual en el aprendizaje por descubrimiento de la cinemática bidimensional en estudiantes de educación media. Edutec Rev. Electrón. Tecnol. Educ..

[22] Barrios Araya S., Urrutia Egaña M., Rubio Acuña M. (2017). Impacto de La Simulación En El Desarrollo de La Autoeficacia y Del Locus de Control En Estudiantes de Enfermería. Educ. Méd. Super..

[23] Caballero M.C., Mariño S.I., López M.V. (2016). Software Para El Aprendizaje de Las Técnicas de Modelado y Simulación. https://www.semanticscholar.org/paper/Software-para-el-aprendizaje-de-las-t%C3%A9cnicas-de-y-Caballero-L%C3%B3pez/c926ffa36cb6a06dc31cb52fa468cff0fd869948.

[24] De la Horra Gutiérrez I. (2010). La Simulación Clínica Como Herramienta de Evaluación de Competencias En La Formación de Enfermería. Reduca.

[25] Valencia J., Tapia S., Olivares S. (2019). La Simulación Clínica Como Estrategia Para El Desarrollo Del Pensamiento Crítico En Estudiantes de Medicina. Investig. Educ. Méd..

[26] Alharbi A., Nurfianti A., Mullen R.F., McClure J.D., Miller W.H. (2024). The Effectiveness of Simulation-Based Learning (SBL) on Students’ Knowledge and Skills in Nursing Programs: A Systematic Review. BMC Med. Educ..

[27] DANE—Estratificación Socioeconómica. https://www.dane.gov.co/index.php/servicios-al-ciudadano/servicios-informacion/estratificacion-socioeconomica.

[28] Eatough V., Smith J.A., Willig C., Stainton-Rogers W. (2017). Interpretative Phenomenological Analysis.

[29] Creswell J.W., Poth C.N. (2016). Qualitative Inquiry and Research Design: Choosing Among Five Approaches.

[30] Fernández-Quiroga M., Yévenes V., Gómez D., Villarroel E. (2017). Uso de La Simulación Clínica Como Estrategia de Aprendizaje Para El Desarrollo de Habilidades Comunicacionales En Estudiantes de Medicina. FEM Rev. Fund. Educ. Méd..

[31] Peñata Ávila A.E., Camargo Zapata E.A., García L.F. (2016). Implementación de Simulaciones Virtuales En La Enseñanza de Física y Química Para La Educación Media En La Subregión de Urabá, Antioquia. Master’s Thesis.

[32] Gaba D.M. (2007). The Future Vision of Simulation in Healthcare. Simul. Healthc..

[33] Matzumura Kasano J.P., León Gamarra H.M., Gutiérrez Crespo H.F. (2018). Simulación Clínica y Quirúrgica En La Educación Médica: Aplicación En Obstetricia y Ginecología. Rev. Peru. Ginecol. Obstet..

[34] Naranjo Rojas A., Cruz Mosquera F.E. (2022). Simulación Clínica En El Aprendizaje de La Técnica de Succión a Través de Traqueostomía. Enferm. Investig..

[35] Arias Guerrero M.A., Sandia Saldivia B.E., Mora Gallardo E.J. (2012). La Didáctica y Las Herramientas Tecnológicas Web En La Educación Interactiva a Distancia. Educere.

[36] Perdomo-Martínez A.M., Díaz-Jurado L.C., Cedeño-Tapia S.J., Escalona-Márquez L.N., Calderón-Padillacon M.C., Villanueva-Rodríguez J.A. (2022). Satisfacción Estudiantil Sobre La Simulación Clínica Como Estrategia Didáctica En Enfermería. Enferm. Investig..

[37] Altamirano-Droguett J.E. (2019). La Simulación Clínica: Un Aporte Para La Enseñanza y Aprendizaje En El Área de Obstetricia. Rev. Electrónica Educ..

[38] Urra Medina E., Sandoval Barrientos S., Irribarren Navarro F. (2017). El Desafío y Futuro de La Simulación Como Estrategia de Enseñanza En Enfermería. Investig. En Educ. Méd..

