# Supplementary material for: Perception of University Nursing Students and Faculty Members Regarding Simulated Practices: A Mixed Methods Study
Source: Nurs Rep. 2024 Oct 14;14(4):2975–89. doi: 10.3390/nursrep14040217 (PMC11503441; doi:10.3390/nursrep14040217)
Supplement: Supplementary file 1 [file nursrep-14-00217-s001.zip › Supplementary File S1.pdf]

**Supplementary File S1. Survey**

## A. Students survey

**Date:** DD MM YYYY

**Specific Objective:** To evaluate the perception of fourth-semester nursing students regarding the factors and elements that influence their performance and learning process in simulated and real practice.

**Sociodemographic Information of Students**

- **Age:** _____________
- **Gender:** Male ( ) Female ( )
- **Civil Status:** Single ( ) Married ( ) Divorced ( ) Free Union ( ) Widow(er) ( )
- **Socioeconomic stratum of residence:** ( )
- **Number of children:** ( )
- **Currently employed:** Yes ( ) No ( )

### SIMULATED PRACTICES

1. Did you participate in simulated practices in the fourth-semester? Yes ( ) No ( )
2. Did you participate in real practices in the fourth-semester? Yes ( ) No ( )
3. In which healthcare institution did you carry out these real practices? _________________
4. Do you consider that you had the adequate theoretical foundation when performing the simulated practice? Yes ( ) No ( )
5. Do you think that the simulated practice contributes to the learning process during the semester? Yes ( ) No ( )

---

### ASSESSMENT OF SIMULATED PRACTICE ASPECTS

Rate from 1 to 4 where:

- **1=**Insufficient; **2=**Sufficient; **3=**Good; **4:** Very Suitable

### Item

- Theoretical foundation
- Length of practice
- Clarity of the clinical cases
- Clarity of procedures
- Teaching support
- Complexity of clinical cases
- Realism of procedures

---

**Specific Objective:** To evaluate the perception of fourth-semester nursing teachers regarding the factors and elements that influence their performance and learning process in simulated and real practice.

The values from 1 to 4 will depend on the context of the question. Below, you will find the opinion or evaluation options according to the meaning of the question or opinion:

**1** = Never; **2** = Rarely; **3** = Almost always; **4** = always

**1** = Insufficient, **2** = Sufficient **3** = Good **4** = Very Suitable

**1** = Not suitable **2** = Inappropriate **3** = Appropriate **4** = Very suitable

## Item

- Do you think that simulated practice contributes to academic performance in the Nursing Care course for early disease detection in the fourth semester?
- Do you think that the topics covered in class were treated in-depth during the simulated practice?
- Do you think that simulated practice allows you to apply the theoretical foundation?
- Do you think the teacher has mastery of the topics during simulated practice?
- Do you think the simulated hospital is sufficiently equipped to meet the course objectives?
- Does the teacher take the necessary time to explain the procedures on the simulation equipment?
- Is the time for each practice enough to develop the procedures?
- Does the teacher allow you to repeat a procedure until you learn it?
- Do you feel the need to repeat a procedure more than once in simulated practice?
- Do you think the simulated practice equipment is similar to the real practice equipment?
- Do you feel your classmates support and collaborate with you to learn during simulated practice?
- Do you think theoretical knowledge is evaluated during simulated practic?
- Do you think what you have learned is adequately evaluated during simulated practice?
- Do you feel that simulated practice efficiently prepares you for real practice?

---

**RATING THE LEVEL OF DEMAND**

Rate the level of demand for the following aspects of simulated practices, where 1 is not demanding at all, and 4 is very demanding.

**Item**

- The methodology used by the teacher
- The clinical cases presented during the practice
- The evaluation methods
- The theoretical foundation for each procedure
- The setting of each activity

## B. Teachers survey

**Date:** DD MM YYYY

**Specific Objective:** To evaluate the perception of fourth-semester nursing teachers regarding the factors and elements that influence their performance and learning process in simulated and real practice.

**Sociodemographic Information of Teachers**

- **Age**: _____________
- **Gender:** Male ( ) Female ( )
- **Civil Status:** Single ( ) Married ( ) Divorced ( ) Free Union ( ) Widow(er) ( )
- **Socioeconomic stratum of residence**: 1 ( ) 2 ( ) 3 ( ) 4 ( )
- **Number of children:** ( )
- **Years of teaching experience:** _______
- **Semester of experience in teaching simulated practices:** _______

### SIMULATED PRACTICES

1. Before leading the simulated practice for fourth-semester nursing students at USC, did you have any experience in teaching in simulated environments? Yes ( ) No ( )
2. Were you trained by the University for the development and teaching of practices in simulated environments? Yes ( ) No ( )
3. Do you currently consider training in the teaching of practices in simulated environments? Yes ( ) No ( )

---

### ASSESSMENT OF SIMULATED PRACTICE ASPECTS

Rate from 1 to 4 where:

- **1=**Insufficient; **2=**Sufficient; **3=**Good; **4:** Very Suitable

### Item

- Theoretical foundation
- Length of practice
- Clarity of the clinical cases
- Clarity of procedures
- Teaching support
- Complexity of clinical cases
- Realism of procedures

---

**Specific Objective:** To evaluate the perception of fourth-semester nursing teachers regarding the factors and elements that influence their performance and learning process in simulated and real practice

The values from 1 to 4 will depend on the context of the question. Below, you will find the opinion or evaluation options according to the meaning of the question or opinion:

**1** = Never; **2** = Rarely; **3** = Almost always; **4** = always

**1** = Insufficient, **2** = Sufficient **3** = Good **4** = Very Suitable

**1** = Not suitable **2** = Inappropriate **3** = Appropriate **4** = Very suitable

## Item

- Do you think the topics covered in the simulated practice were addressed in depth?
- Do you think simulated practice helps students master concepts?
- Do you think simulated practice helps students understand procedures?
- Do students achieve mastery of topics during simulated practice?
- Do you find the simulated practice environment realistic?
- Is there enough time to explain the use of simulation equipment?
- Is the time used in each practice sufficient for the development of procedures?
- Are the number of practices scheduled during the semester enough to integrate knowledge?
- Does the time and structure of the simulated practice allow students to repeat a procedure until it is fully learned?
- Do you feel the need to repeat a procedure more than once in simulated practice?
- Do you think the simulated practice equipment is similar to the real practice equipment?
- Do you feel that students support and collaborate with each other to learn in simulated practice?
- Do you think the assessments in simulated practice are demanding?
- Do you think the exams and tests adequately evaluate what has been learned?
- Do you think simulated practice prepares students efficiently for real practice?
- Is the number of students per teacher adequate to allow repetition of procedures?

---

**RATING THE LEVEL OF DEMAND**

Rate the level of demand for the following aspects of simulated practices, where 1 is not demanding at all, and 4 is very demanding.

**Item**

The methodology used by the teacher

- The methodology used
- The clinical cases presented during the practice
- The evaluation methods
- The theoretical foundation for each procedure
- The setting of each procedure
