# Supplementary material for: Perception of University Nursing Students and Faculty Members Regarding Simulated Practices: A Mixed Methods Study
Source: Nurs Rep. 2024 Oct 14;14(4):2975–89. doi: 10.3390/nursrep14040217 (PMC11503441; doi:10.3390/nursrep14040217)
Supplement: Supplementary file 1 [file nursrep-14-00217-s001.zip › Supplementary File S2.pdf]

Supplementary File S2. Semi-structured interview guide

A. Questions for students

Question	Description
1. How do you rate the level of realism in the simulated practices?	The aim is to understand the student's perception of the similarity between simulated and actual practices.
2. Do you think simulated internships adequately prepare you for real internships?	It investigates the preparation that students feel when facing real clinical situations.
3. What aspects would you improve in simulated practices?	The objective is to learn students' suggestions to improve the simulation process.
4. How do you perceive the teacher's support during the simulations?	The guidance and support received from the teacher during the internship are evaluated.
5. Do you consider that the time dedicated to simulations is sufficient?	Questions are asked about the adequacy of the time allocated for the simulated practices.

B. Questions for teachers

Question	Description
1. What is your opinion on the usefulness of simulated practices in the training of students?	The aim is to understand teachers' perceptions of the value of simulations in student learning.
2. What are the main challenges you encounter when implementing simulated practices?	The difficulties faced by teachers in the simulation process are investigated.
3. How would you rate the equipment and simulators' realism?	The question is about the teacher's perception of the quality and realism of the equipment used in the simulations.
4. Do you consider the time allocated for the simulations adequate?	Teachers' perception of the amount of time spent on simulations is assessed.
5. What changes would you implement to improve the effectiveness of simulated practices?	Suggestions for improvement based on the teacher's experience are investigated.
